# Adaptional evolution of trichome in *Caragana korshinskii* to natural drought stress on the Loess Plateau, China

**DOI:** 10.1002/ece3.2157

**Published:** 2016-05-05

**Authors:** Pengbo Ning, Junhui Wang, Yulu Zhou, Lifang Gao, Jun Wang, Chunmei Gong

**Affiliations:** ^1^ College of Life Science Northwest A&F University Yangling Shaanxi 712100 China; ^2^ School of Life Science and Technology Xidian University Xi'an Shaanxi 710071 China

**Keywords:** *Caragana korshinskii*, drought, real‐time PCR, transcriptome analysis, trichome

## Abstract

*Caragana korshinskii* is commonly employed to improve drought ecosystems on the Loess Plateau, although the molecular mechanism at work is poorly understood, particularly in terms of the plant's ability to tolerate drought stress. Water is the most severe limiting factor for plant growth on the Loess Plateau. The trichome is known to play an efficient role in reducing water loss through decreasing the rate of transpiration, so in this study, we focused on the trichome‐related gene expression of ecological adaptation in *C. korshinskii* under low precipitation conditions. In order to explore the responses of trichomes to drought, we selected two experimental sites from wet to dry along the Loess Plateau latitude gradient for observation. Micro‐phenomena through which trichomes grew denser and larger under reduced precipitation were observed using a scanning electron microscope; *de novo* transcriptomes and quantitative PCR were then used to explore and verify gene expression patterns of *C. korshinskii* trichomes. Results showed that *GIS2*,*TTG1*, and *GL2* were upregulated (as key positive‐regulated genes on trichome development), while *CPC* was downregulated (negative‐regulated gene). Taken together, our data indicate that downstream genes of gibberellin and cytokinin signaling pathways, alongside several cytoskeleton‐related genes, contribute to modulating trichome development to enhance transpiration resistance ability and increase the resistance to drought stress in *C. korshinskii*.

## Introduction

Precipitation is, across the globe, decreasing due to climate change (Xu et al. 2006; Zhang et al. 2010; Eslamian et al. 2011) especially in arid and semi‐arid regions in China (Piao et al. 2010). Water deficiency is the most severe limiting factor for plant growth, development, and reproducibility (Jaleel et al. 2009). Subjected to dry climate and soil moisture‐deficit conditions, the Loess Plateau faces sizable challenges in terms of vegetation degradation and weaker ecosystem stability (Vägen et al. 2005; Yan et al. 2010; Kim et al. 2013; Alam et al. 2013; Yang et al. 2014). *Caragana korshinskii*, which shows naturally high tolerance to drought stress, is commonly artificially planted in the Loess Plateau in effort to protect the ecosystem from desertification (Wang et al. 2007).

The trichome, a specialized structure in plant epidermal cells, is typically long and dense in morphological characteristics in xeromorphic plants (Gianoli and González‐Teuber 2005). Many previous studies have focused on examining trichome characteristic responses to soil water deficit in effort to determine whether (and to what extent,) the trichome enhances drought resistance ability in plants (Gianoli and González‐Teuber 2005; Huttunen et al. 2010; Meng et al. 2014). Trichome density is likely a plastic adaptive pattern to drought, on account of its barrier effect against the influence of CO_2_ and H_2_O exchange, which reduces excessive transpiration and photoinhibition (Pallioti et al. 1994; Gianoli and González‐Teuber 2005; Fu et al. 2013). Trichomes also can reduce the plant's solar radiation absorption and decrease its temperature by increasing the leaf surface boundary layer, further protecting the plant from drought (Schreuder et al. 2001).

*Tamarix chinensis* can discharge salt in liquid state with trichomes, allowing the plant to grow normally in saline alkali soil. In some species of *Aizoaceae* and *Chenopodiaceae*, the trichome acts as one type of water‐storage tissue, they play a role of water storage by controlling the smallest surface area (circular) and high concentrations of cell liquids to keep transpiration slow. Tetraploid black locust has shown stronger drought resistance with higher adaxial trichome density, similarly to *Arabidopsis lyrata* and *Arabidopsis kamchatica* (*Brassicaceae*) (Steets et al. 2010; Sletvold and Ågren 2012; Meng et al. 2014).

Researchers have identified the rough molecular basis and regulatory networks of trichome growth and development (Esch et al. 2004; Serna and Martin 2006). Major regulators of trichome development include positive and negative regulators. The positive regulators consist of three protein classes: R2R3 MYB‐related transcription factors, basic helix‐loop‐helix (bHLH)‐like transcription factors, and a WD40 protein TRANSPARENT TESTA GLABRA1 (TTG1). MYB‐bHLH‐WD40 complex combines the promoter GLABRA2 to activate the trichome reaction, and several C2H2 transcription factors act upstream of GL1 to regulate trichome initiation (Gan et al. 2007; Zhou et al. 2013). The negative regulators include several single‐repeat R3 MYBs (Wang et al. 2008). For lacking the C‐terminal activation domain, the negative regulators such as CAPRICE (CPC), TRIPTYCHON (TRY), TRICHOMELESS1 (TCL1), enhancer of TRY, and CPC 1, 2, and 3 (ETC1, ETC2, and ETC3) suppress trichome initiation by replacing the R2R3 MYB‐related transcription factors of the activator complex (Ohashi et al. 2002). The interaction of these positive and negative regulators results in the initiation and development of trichomes.

An investigation was found that trichomes of *C. korshinskii* both in leaf adaxial and abaxial sides are densely clustered in the area with more serious water‐deficit conditions; however, the mechanism that trichomes of *C. korshinskii* become denser and larger under drought conditions needs more attention to clarify. How does the trichome of *C. korshinskii* play an efficient role to resist drought stress is unconcern yet. In this study, we examined samples of *C. korshinskii* distributed throughout Huangling and Dalad Banner woodlands (i.e., wet and dry experimental sites) along the latitude gradient of the Loess Plateau. We investigated differences in *C. korshinskii* trichome phenotype and its regulatory mechanism via transcriptome analyses and real‐time PCR. Our primary goal was to answer the following questions: (i) how does the distribution rule of *C. korshinskii* trichomes in both adaxial and abaxial leaves change in response to drought? (ii) how do the changes of several trichome‐related genes in *C. korshinskii* respond to reduced precipitation? and (iii) how does the regulatory mechanism of trichomes in *C. korshinskii* as a widely distributed species adapt to harsh abiotic environments in arid and semi‐arid regions of China?

## Materials and Methods

### Plant materials and growth conditions

**Figure 1:**
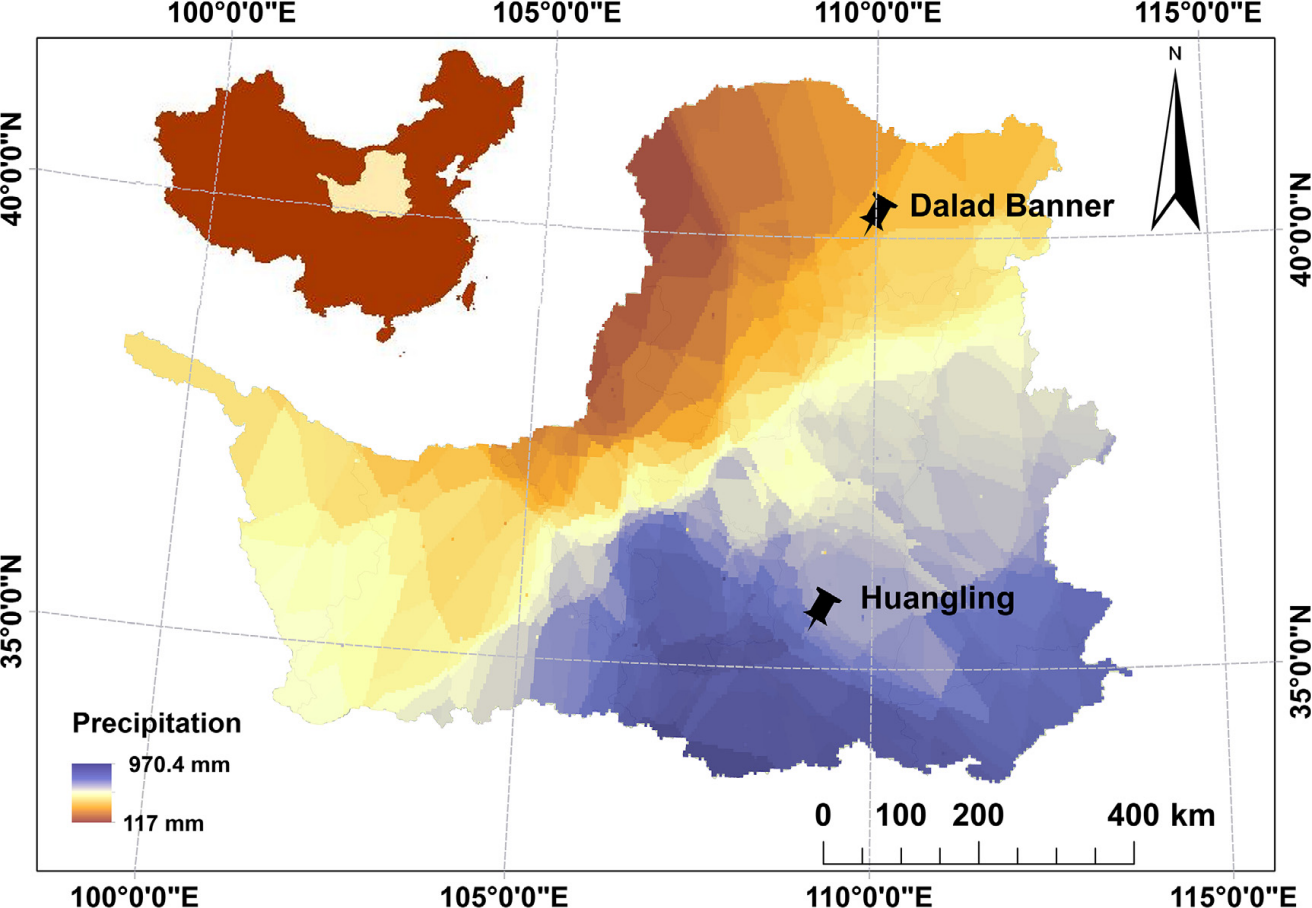
Experimental sites for *Caragana korshinskii* along the Loess Plateau precipitation reduction.

*Caragana korshinskii* leaves were sampled as experimental materials in mid‐July 2014, and the ages of different *Caragana korshinskii* shrublands were all over 20 years. Several fresh braches were picked from at least nine representative shrublands, and health leaves that were selected from branches were mixed and collected in perforated centrifuge tubes (5 mL); then, the tubes were marked and placed in liquid nitrogen container immediately, and then kept at −80°C in the laboratory. Several fresh leaves (5 mm×5 mm) were also selected to fix in 4% glutaraldehyde and preserved in ice boxes, then kept at 4°C in the laboratory. Huangling and Dalad Banner were chosen as experimental sites along the precipitation reduction of the Loess Plateau in Northwest China (Fig. 1). Huangling is located in the south of Loess Plateau (35°39′N 109°14′E) with 578.7 ± 8.8 mm annual precipitation, while Dalad Banner is in the north of Loess Plateau (40°14′N 109°58′E) with 311.6 ± 4.5 mm annual precipitation, other conditions such as the annual temperature, altitude, and soil pH of two sites does not have a significant difference. From Huangling to Dalad Banner, the increase in aridity index is in accordance with the decrease in annual precipitation shown in Table 1, so Huangling and Dalad Banner were chosen as material sites to represent the different soil water conditions. Experimental sites we selected were natural and far away from downtown to avoid the interference of human activity; *Caragana korshinskii* acts as a dominant species and auxiliary species including *Hedysarum scoparium*,* Haloxylon ammodendron,* and another adversity‐resistant desert plants in sites.

**Table 1:** Geographical profiles of experimental sites chosen in this study

Experimental sites	Location	Annual precipitation (mm)	Annual evapotranspiration (mm)	Soil moisture content (%)	Annual temperature (°C)	Soil pH	Altitude (m)
Huangling	35°39′N 109°14′E	578.7 ± 8.8	1323 ± 136	10.4 ± 0.9	9.1 ± 1.5	6.5	1050
Dalad Banner	40°14′N 109°58′E	311.6 ± 4.5	2168.3 ± 28.7	0.4 ± 0.1	7.5 ± 0.5	6.3	1150

### Scanning electron microscopy

We used a scanning electron microscope (S‐3400N, Hitachi, Japan) to observe the arrangement trichomes on our *C. korshinskii* leaf samples. According to methods outlined by Chen et al. (2008), leaf samples (5 mm × 5 mm) were fixed in 4% glutaraldehyde at 4°C for over 6 h, and then washed three times with 0.1 M phosphate buffer solution for 10 min. The samples were then transferred into 1% osmic acid at 4°C for 2 h and washed three times with 0.1 M phosphate buffer solution for 10 min. The samples were then dehydrated with a series of ethanol (30%, 50%, 70%, 85%, and 100%) mixtures for 20 min, respectively, followed by a series of tert‐butyl alcohol (50%, 75%, 100%) twice to remove the ethanol. After being dried in a freeze drying box (VFD‐21S) at −40°C overnight, samples were sprayed with a 12.5–15 nm gold layer and examined/photographed in different multiperspectives with the scanning electron microscope.

### Digital transcriptomics

To investigate and compare the complex biological processes induced across wet and dry experimental sites as the different precipitation, a *de novo* transcriptome analysis (based on Solexa sequencing) was designed to investigate gene expression (Exposito‐Rodriguez et al. 2008). We used the total RNA (1–10 mg) of *C. korshinskii* leaves from Huangling and Dalad Banner to construct each *de novo* library, and Cluster and Java Treeview to establish similar gene expression patterns via cluster analyses of gene expression patterns (Gibbons and Roth 2002; Ning et al. 2014).

### Construction of the phylogenetic tree

We used clustalx‐2.1‐win.msi to analyze the sequence of *TTG1* reported in some plants downloaded from NCBI (http://www.ncbi.nlm.nih.gov). The Bayesian phylogenetic tree based on gene *TTG1* was constructed with MrBayes version 3.1.2 (University of California, San Diego, CA, USA).

### Real‐time PCR

The critical genes related to trichome development, *TTG1*,* CPC*,* GIS2*, and *GL2*, were selected for real‐time PCR amplification. *TTG1*‐specific primers, *TTG1*‐S (5ʹ‐ATCCATCAGCGGAAACGG‐3ʹ) and *TTG1*‐AS (5ʹ‐GCAATCTCCCTCTTCAACAACAG‐3ʹ), were used to amplify the 235‐bp‐long region, and *CPC*‐specific primers, *CPC*‐S (5ʹ‐TACGACCTGGAATGCGACC‐3ʹ) and *CPC*‐AS (5ʹ‐CCCTCCGTTGTTTTCATAAGC‐3ʹ), were used to amplify the 217‐bp‐long region. *GIS2*‐specific primers, *GIS2*‐S (5ʹ‐GATGGCAAGTTATGGGTTATGGT‐3ʹ) and *GIS2*‐AS (5ʹ‐TCCTTACGAAAAATAGCCTAATCAG‐3ʹ), were used to amplify the 253‐bp‐long region, and *GL2*‐specific primers, *GL2*‐S (5ʹ‐TTTAGCCAAAGGACAAGACCG‐3ʹ) and *GL2*‐AS (5ʹ‐GCTTGAATCACATCCAGTCATAAC‐3ʹ), were used to amplify the 175‐bp‐long region. We run real‐time PCR as follows: one cycle at 94°C for 30 s and 40 cycles of denaturation at 94°C for 5 sec, annealing 58°C for 30 sec, and final extension at 72°C for 5 min. We used a BIO‐RAD iQ5 Multicolor Real‐Time Detection System (Bio‐Rad, Hercules, CA, USA) to perform all real‐time PCR reactions and determined the relative expression of the tested reference genes by CT values calculated by 2^−ΔΔCt^ method (Wong and Medrano 2005).

### Statistical analyses

The present study has calculated the density and the size of leaf trichomes and analyzed the expression level of critical genes of trichome development of Huangling and Dalad Banner. The data of latitude and longitude and average annual precipitation of the material sites (Huangling and Dalad Banner) were collected using ArcGIS 10.1 (Environmental Systems Research Institute Inc., Redlands, CA, USA). The density and the size of leaf trichomes were calculated from SEM pictures, the size was measured by Image J 2X, and data were presented as the mean standard error (SE) of at least six replicate tests. The result of RT‐PCR of *TTG1*,* GL2*,* GIS2,* and *CPC* was obtained using SPSS 17.0 (SPSS Inc., Chicago, IL, USA) after CT values was calculated by 2^−ΔΔCt^ method; one‐way ANOVA was used to analyze significance tests, and Origin 9.0 was chosen to process the above data.

## Results

### *Caragana korshinskii* leaf trichome development

**Figure 2:**
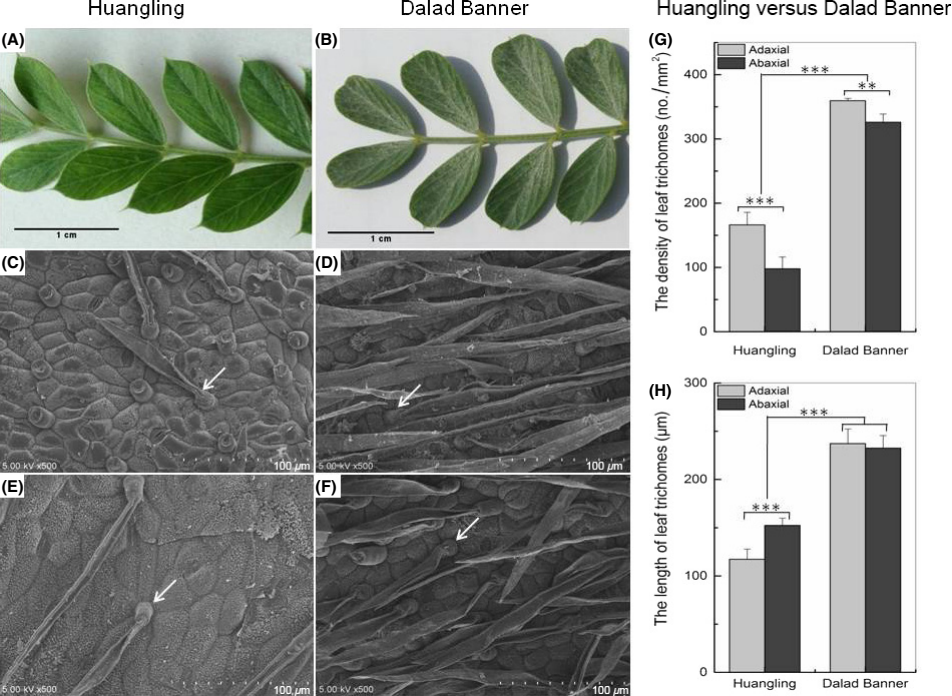
Leaf epidermis trichome of *Caragana korshinskii* in different habitats observed under the camera and scanning electron microscope. (A) Adaxial surface in Huangling. (B) adaxial surface in Dalad Banner. (C) adaxial surface in Huangling ×500. (D) adaxial surface in Dalad Banner ×500. (E) abaxial surface in Huangling ×500. (F) abaxial surface in Dalad Banner ×500. (G) variation trend of trichome density. (H) variation trend of trichome length. ** indicates significance (*P *<* *0.01), *** indicates high significance (*P *<* *0.001).

Differences in trichome morphological characteristics on the surfaces of the leaves from the two experimental sites are shown in Figure 2(A and B). To characterize the morphology of *C. korshinskii* trichomes in detail, as discussed above, we observed the adaxial and abaxial trichomes of *C. korshinskii* from Huangling and Dalad Banner using SEM. The abundance of adaxial leaf trichomes between the two sites differed considerably as well as abaxial leaf trichomes due to differing precipitation levels, and the density and the size of both adaxial and abaxial trichomes of Dalad Banner plants were larger than those of Huangling plants (Fig. 2C–F).

The densities of adaxial and abaxial trichomes of Dalad Banner plants were 359 ± 3.54 and 326 ± 12.62 no. per mm^2^, respectively, while those of Huangling trichomes were 166 ± 19.55 and 98 ± 18.25 no. per mm^2^ (Fig. 2G). Basically, trichome density increases as precipitation decreases, with very high statistical significance (*P *<* *0.001). Similar phenomena appeared in terms of trichome length – the lengths of adaxial and abaxial trichomes of Dalad Banner plants were 237 ± 15.07 *μ*m and 233 ± 12.96 *μ*m, while those of Huangling plant trichomes were 117 ± 10.46 *μ*m and 152 ± 7.36 *μ*m (Fig. 2H), and again, the difference was highly statistically significant (*P *<* *0.001). In short, the *C. korshinskii* trichome appears denser and longer in water‐deficient environments than in environments with more stable precipitation.

### *Caragana korshinskii* trichome transcriptional profiling via DEG (transcriptome‐Differentially Expressed Gene deep analysis)

**Figure 3:**
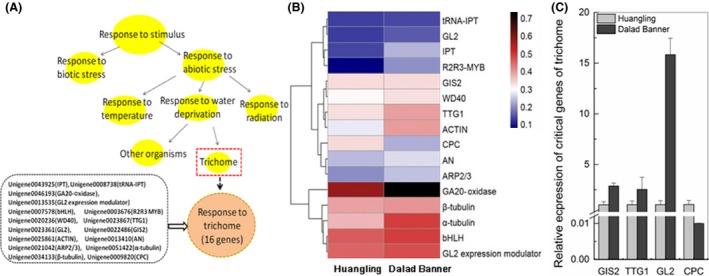
The trichome, an organism which responds to water deprivation and expression level of critical genes involved in its initiation and development. (A) Trichome acts as a drought‐resistant tissue. (B) expression level of trichome development‐related genes of Huangling vs. Dalad Banner. (C) relative expression of critical trichome development genes of Huangling vs. Dalad Banner.

To identify genes potentially associated with trichome development related to abiotic stress, we used a *de novo* transcriptome analysis based on Solexa sequencing to explore the *C. korshinskii* samples from Huangling and Dalad Banner. Results showed that several key genes of trichome development were enriched in the “response to stimulus” of the significant GO term (*P *<* *0.05, Fig. 3A) after Gene Ontology (GO) analysis, which showed upregulated or downregulated genes according to different precipitation (Fig. 3B). Upregulated genes included *TTG1*,* GL2*,* GIS2*,* AN*,* ARP2/3*, and others; only *CPC* was downregulated, suggesting that it is a negative regulator of trichome development. The rates of gibberellin (GA) and cytokinin (CTK) enzyme limitation, including GA‐20 oxidas, tRNA isopentenyl‐transferase (tRNA‐IPT), and adenylate isopentenyl‐transferase (IPT), also were upregulated. We submitted the same samples from Huangling and Dalad Banner to real‐time PCR amplification and found, as shown in Figure 3(C), that the expressions of *GIS2*,* TTG1,* and *GL2* were significantly upregulated and expression of the negative regulator *CPC* was downregulated in Dalad Banner compared to Huangling.

### The phylogenetic relationships among *Caragana korshinskii* and other species by *TTG1*

**Figure 4:**
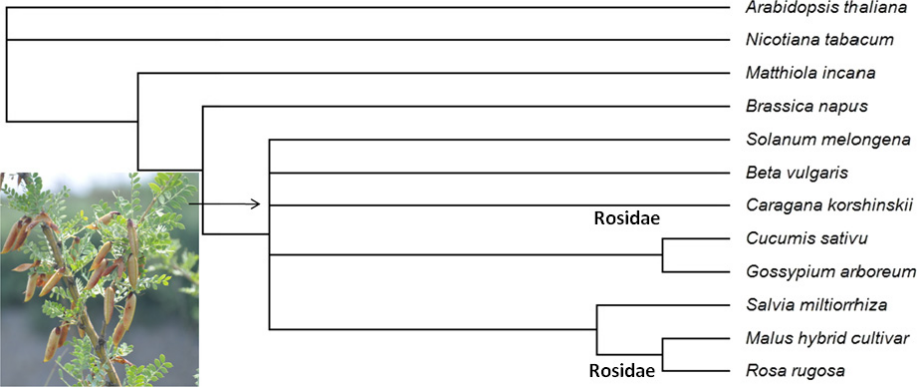
The phylogenetic relationships were indicated among *Caragana korshinskii* and some plants which *TTG1* is working related to trichome development.

We run the phylogenetic tree according to the *TTG1* senquences of plants submitted to NCBI and *TTG1* sequences of *Caragana korshinskii* we have (Fig. 4), although the evolution of *Caragana korshinskii* was not concluded clearly, just closer to *Gossypium arboretum*,* Salvia miltiorrhiza,* and some vegetables. But, it belongs to legumes in Rosidae, which is close to *Rosa rugosa* in the same subclass, so we might get some inspiration and similarities from the close species *Rosa rugosa* in Bayesian phylogenetic relationships.

## Discussion

### The role of leaf trichome in *Caragana korshinskii*

In order to adapt to water deficit, plants that grow under drought conditions need certain physiological mechanisms to hold enough water to maintain normal physiological metabolism. Plants lose most water due to transpiration, the rate of which is affected considerably by diffusion resistance (Lange et al. 1971; Pangle et al. 2015). The trichome is a specialized structure of leaf epidermal cells that covers the plant's surface and serves to enhance its diffuse resistance to transpiration (Fu et al. 2013; Westberg et al. 2013). In this study, the *C. korshinskii* trichome was observed to be larger and denser due to the plant's necessity for slowed transpiration rate, and, as such, contributed effectively to enhancing the plant's drought resistance (Fig. 2).

### The regulatory mechanism of trichomes in *C. korshinskii*

The molecular basis and regulatory networks of trichome growth and development have already been elucidated (Esch et al. 2004; Serna and Martin 2006; Taheri et al. 2015). GA and CTK pathways have been reported extensively to illuminate the regulation mechanism of trichome development, and the biosynthesis of CTK and GA may result from the upregulation of rate‐limited enzymes (Ogas et al. 1997; Gan et al. 2007; Zhao et al. 2008). GA is known to accelerate cell division and longitudinal elongation, while CTK can accelerate cell division and transverse elongation – increasing content of either accelerates the differentiation of epidermal cells, so they can be attributed to trichome formation (Blackwell and Horgan 1994; Åstot et al. 2000; Golovko et al. 2002). The cytoskeleton pathway has also been shown to play an essential role in the normal growth and development of trichomes (Folkers et al. 2002; Kim et al. 2002).

Digital transcriptome sequencing analysis showed that several critical genes and regulators of trichome development were upregulated or downregulated (Fig. 3B). GA and CTK, well‐known hormones, are crucial for trichome development, proved important – *GIS2* in particular was modulated by the GA and the CTK signaling pathways changed significantly in Dalad Banner relative to Huangling. These observations suggest that enhanced activation of the GA and CTK pathways may be related to enhanced drought stress resistance. The downstream genes of *GIS2* (including *TTG1*,* GL2*, and *CPC*) also changed significantly between the two sample sites.

*GIS2* acts in downstream of GA and CTK to encode C2H2 transcription factors to control trichome development through CTK and GA signaling. Because *GIS2* is a homologue of *GIS*, they can be encoded with the same functional protein (Gan et al. 2007). The downstream gene of *GIS*, the TTG1‐bHLH‐MYB regulatory complex, must be further examined to determine whether it acts in the *GIS2* downstream.

TTG1, a WD40 repeat protein, has been localized in the nuclei of trichomes at all developmental stages in *Arabidopsis* leaves; a lack of trichomes may be a result of TTG1 loss (Walker et al. 1999; Zhao et al. 2008). Studies have shown that TTG1 and R2R3 MYB physically interact with bHLH, but not with each other, and that TTG1 is necessary for the bHLH family protein GL3 to function (Payne et al. 2000; Zhang et al. 2003). Upregulation of *TTG1* in the GA and CTK pathways can promote the expression of downstream genes such as *GL2*. *GL2*, a homeodomain (HD‐Zip) transcription factor, acts downstream of the TTG1‐bHLH‐MYB complex to encode a homeodomain protein related to endoreduplication and maturation of cell walls required for subsequent phases of trichome morphogenesis. As such, upregulation of *GL2* can accelerate trichome formation (Fyvie et al. 2000; Ohashi et al. 2002; Hauser 2014).

Disruption of the TTG1‐bHLH‐MYB complex may be a result of the single‐repeat R3‐MYB protein CPC, negatively regulating trichome development by competing with R2R3 MYB for binding to bHLH. This would inhibit the formation of trichomes by repressing the expression of GL2, so in other words, *CPC* downregulation relieve the inhibition of the differentiation of nonhair cells (Wada et al. 2002; Bernhardt et al. 2003; Kurata et al. 2005; Ishida et al. 2008).

Several critical genes in the cytoskeleton pathway, including actin, actin‐related protein 2/3(ARP2/3), angustifolia (AN), alpha tubulin, and beta tubulin, also were upregulated in our samples. The main composition of microfilament is actin, which is important because the mutation of microtubules or actin microfilaments results distorted and short (i.e., less effective) trichomes (Mathur et al. 1999). Microtubule function is required for normal trichome development, and alpha tubulin and beta tubulin can be integrated effectively into the dimer (which is used as a subunit of microtubule assembly). AN is involved in any high concentration of microtubules at the tip of trichome cells (Folkers et al. 2002; Kim et al. 2002), and the ARP2/3 complex is known to promote the formation of actin networks by initiating the polymerization of new actin filaments around already existing actin (Mullins et al. 1998; Svitkina and Borisy 1999).

**Figure 5:**
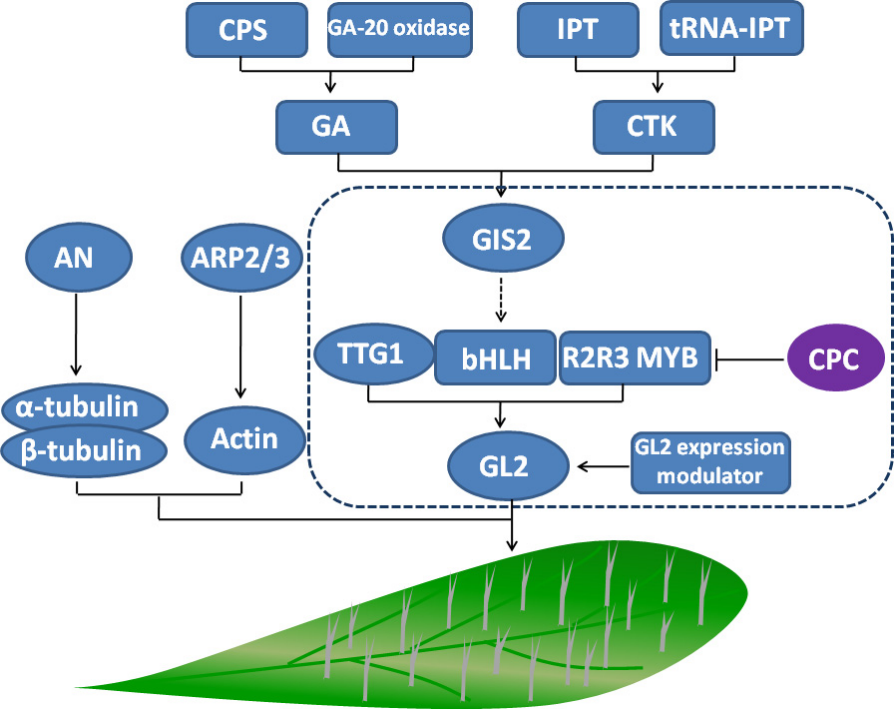
Gibberellin and cytokinin network pathway and cytoskeleton pathway promote trichome initiation and development to enhance *Caragana korshinskii* drought resistance.

As discussed above, under drought conditions, *C. korshinskii* trichomes become denser and bigger to adapt to the living environment. The mechanism model of the trichome gene network as it enhances drought resistance is shown in Fig. 5. GA and CTK pathways play an important role in this process; the expression of rate‐limited enzymes of GA and CTK may contribute to increased GA and CTK content. *GIS2*, for example, was modulated by GA and CTK signaling pathways, and downstream gene TTG1‐bHLH‐MYB complex modulated the expression of GL2, which is affected by CPC competing with R2R3 MYB for binding to bHLH. Our real‐time PCR data for *GIS2*,* TTG1*,* GL2*, and *CPC* were in accordance with our hypotheses that upregulation of *TTG1* modulates the expression of *GL2*, and that upregulation of *GL2* encodes a homeodomain protein to attend to endoreduplication and maturation of the cell walls required for subsequent phases of trichome morphogenesis. We also confirmed that downregulation of *CPC* relieves any inhibited trichome initiation. In short, we confirmed that the network of critical genes we observed do indeed contribute to trichome formation in *C. korshinskii*. In addition, because upregulation of ARP2/3, actin, and AN are crucial for normal trichome development, we found that GA and CTK‐signaling pathways, downstream genes, is cytoskeleton‐related genes together promoted the initiation and development of trichomes to enhance the diffuse resistance to transpiration and increase the plant's ability to live in water‐deficit conditions.

### The phylogenetic relationships among *Caragana korshinskii* and other species by *TTG1*

*TTG1*, a related gene to trichome development regulation, is mainly concentrated in model plants in Dicotyledoneae such as *Arabidopsis thaliana* and *Nicotiana tabacum*, and ornamental plants with apparent trichome phenotype such as *Matthiola incana*. In addition, it was reported in several vegetables such as *Brassica napus*,* Solanum melongena,* and *Cucumis sativu*. However, the research on *TTG1* of trichomes of adversity‐resistant desert plants has not been reported. *C. korshinskii*, a shrubs species in Caragana Fabr., acts as a desert plant with strong ability of drought resistance; the phylogenetic analysis of *TTG1* of *C. korshinskii* may contribute to the analysis of the resistance mechanism of other desert plants.

## Conclusion

The architecture of critical genes involved in trichome initiation and development in *C. korshinskii* plays an indispensable role in the plant's drought resistance properties. Furthermore, denser and larger trichomes that result from appropriate genetic architecture are of critical significance for the survival of plants that grow in drought‐prone environments such as the Loess Plateau and can potentially be controlled to mitigate ecological reconstruction of degraded ecosystems.

## Conflict of Interest

None declared.
